# Exploring Bioactive Polysaccharides in Edible Fruits: A Cross-Biome Perspective

**DOI:** 10.3390/plants14223515

**Published:** 2025-11-18

**Authors:** Karen Rebouças Nascimento, Leandro Teodoro Júnior, Mari Cleide Sogayar, João Paulo Fabi

**Affiliations:** 1 Department of Food Science and Experimental Nutrition, School of Pharmaceutical Sciences, University of São Paulo, São Paulo 05508-000, SP, Brazil; karenscmnto@usp.br; 2 Food Research Center (FoRC), CEPID-FAPESP (Research, Innovation and Dissemination Centers, São Paulo Research Foundation), São Paulo 05508-080, SP, Brazil; 3 Biochemistry Department, Chemistry Institute, University of São Paulo, São Paulo 05508-900, SP, Brazil; teoolt.bio@gmail.com (L.T.J.); mcsoga@iq.usp.br (M.C.S.); 4 Cell and Molecular Therapy NUCEL Group, School of Medicine, University of São Paulo, São Paulo 01246-903, SP, Brazil

**Keywords:** bioactive polysaccharides, carbohydrates, edible fruits, structural composition, biome

## Abstract

The present work consisted of a comparative analysis, followed by an extensive narrative literature review, of the structural profiles of bioactive polysaccharides from edible fruits representing different terrestrial biomes, relating them—with a focus on their monosaccharide fractions—to the abiotic variables of each biome, such as temperature, rainfall, annual water regimes, and physicochemical characteristics of the soil to provide an accurate landscape regarding the patterns and divergences surrounding the development of edible fruits around the world. The present review also provided a focus on the various analytical methods used to obtain data related to the glycosidic profile of the analyzed edible fruits, allowing for a comparison of issues relating to the biomes and the quantitative composition of the existing polysaccharides, together with the associated macromolecular parameters, such as degree of esterification, branching, and average molecular weight. From the analysis performed, recurrences of characteristics were identified in different biomes, such as high concentrations of galacturonic acid and arabinose in fruits from cold regions; abundance of xyloarabinan and galactan in fruits from arid areas; and greater branching, acetylation, and a lower degree of esterification in fruits subject to water variations that favor water retention and cell wall stability. These profiles suggest a strong association between the structure of polysaccharides and ecological adaptations that are crucial for their full development. The insights presented here are of the utmost importance in both basic and applied food science, indicating possible structural targets for selecting and engineering resistance in edible fruits under various abiotic stress conditions and guiding and providing direction for experimental studies that extend beyond classical methodologies.

## 1. Introduction

Polysaccharides are intricate carbohydrates with varied functions in nature. They are structural and protective components in plants, but they also provide important health benefits to humans [1]. Such forms, including pectins, arabinogalactans, galacturonans, and other structural carbs, denominated as bioactive polysaccharides, are prevalent in edible fruits and have prebiotic, antioxidant, and immunomodulatory activities [2,3,4].

Polysaccharides play complex physiological roles in fruits, from providing structural support to acting as energy storage and facilitating cell recognition and signaling [5,6]. They are strategically distributed within fruit tissues in quantities that reflect their functional priorities [7]. Differences between species, developmental stages (e.g., fruit maturation), and environmental factors, such as soil characteristics and their physical-chemical variations, temperature, and precipitation regimes within the native biome, are responsible for variations in polysaccharide composition [8,9].

The Earth’s biomes show significant spatial heterogeneity in their structural and climatic conditions. Tree ecosystems range from high, dense forests of megaphyllous plants to open shrublands with xeromorphic vegetation [10]. Desert biomes exhibit extreme thermal oscillations, characterized by diel or seasonal temperature fluctuations ranging from subzero minima to maxima exceeding 50 °C [11]. Hydrological regimes also differ, from hyper-humid regions with >2000 mm annual precipitation to arid regions with <100 mm [12]. Despite the differences in abiotic factors, such as temperature, rainfall, and soil, angiosperm taxa have developed fruit-forming mechanisms that span every prominent terrestrial biome, facilitating the worldwide distribution of edible reproductive structures [13].

Global variation in biomes, each with its characteristic geographical and climatic conditions, creates significant diversity among fruits in terms of morphology, flavor profiles, aroma volatiles, and biochemical composition [14]. The structural functions and functional diversity of the fruits are greatly determined by the environmental conditions of the biomes in which they are found. For instance, the Biome of Tundra fruits synthesize cryo-protective polysaccharides to tolerate freezing conditions [15], and desert-dwelling species synthesize hydrophilic mucilages to tolerate drought [16]. Although these plants are of ecological and nutritional significance, their biome-specific stressors influence the polysaccharide profile, which remains largely unstudied.

These bioactive polysaccharides are biological macromolecules formed by chains of monosaccharides, glycans, linked by α- and β-type glycosidic bonds [17]. They have heterogeneous and predominantly branched structures, associated with diverse biological functions, particularly in signaling and cell recognition processes [17,18].

Their unique molecular structure, combined with other physicochemical characteristics such as molecular weight, degree of branching, and glycan composition, significantly modulate their interaction with the human body and its resident microbiota, allowing for fine-grained control over their bioactivity [19]. The composition of bioactive polysaccharides in fruits is closely related to their ecological and physiological functions, ranging from mechanical resistance to the regulation of permeability and hydration.

**Figure 1:**
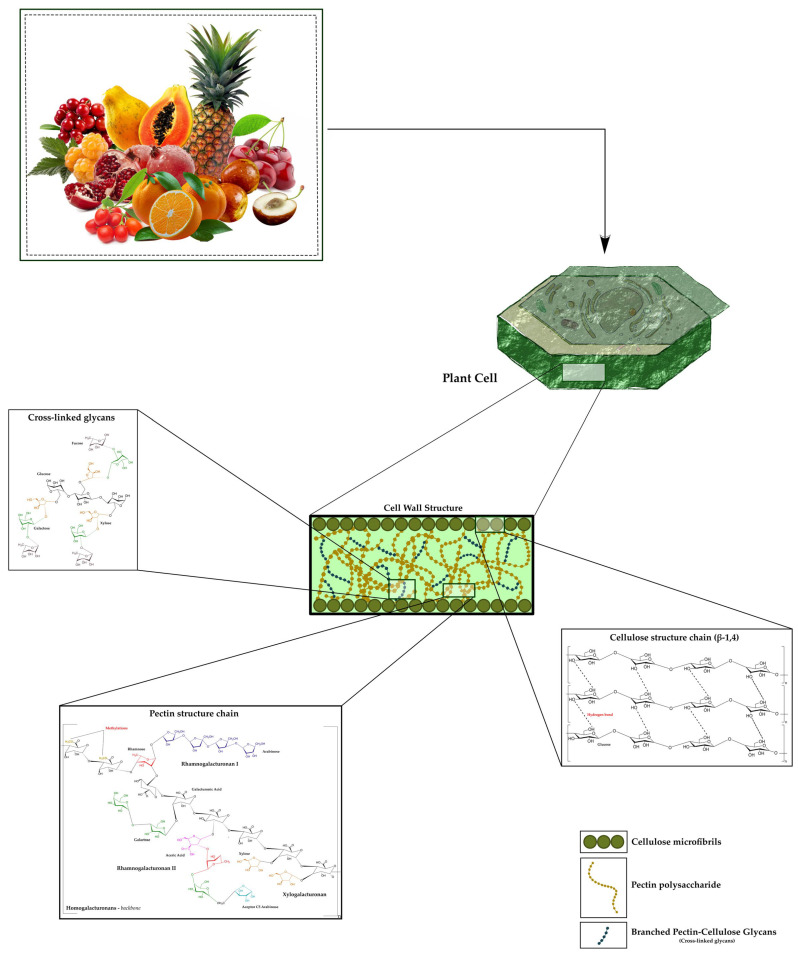
Plant cell wall molecular structure of edible fruits, focused on the major glycans. Drawings generated by the ChemDraw v. 23.1.2, Inkscape v. 1.4 software, and BioArt (NIH) suite online.

The occurrence of bioactive polysaccharides is not restricted to the Plantae domain; however, these are the ones most commonly ingested in the human diet, especially those found in edible fruits. Most bioactive polysaccharides from edible fruits contain glycans, such as glucose (Glc), fucose (Fuc), mannose (Man), galactose (Gal), arabinose (Ara), rhamnose (Rha), and galacturonic acid (GalA), among others [20,21]. They are structurally linked through various glycosidic bonds. The bioactive polysaccharides of edible fruits are mainly made up of neutral sugars like fructose (Fru), Glc, and Gal, which provide the structural framework for both homopolysaccharides and heteropolysaccharides (Figure 1) [20,21,22].

Glc is the predominant component of storage polymers like starch, whereas Gal is commonly found in branched chains of cell wall polysaccharides. The uronic acids, specifically glucuronic and GalA, are found in heteropolysaccharides, such as pectins [23,24]. The acidic chains carry negative charges, enabling the chains to trap cations (Ca^2+^) and form a gel, which is very important for the fruit’s texture. The neutral to acidic sugar ratio varies with fruit maturity and species. For instance, pectins from apples are rich in GalA content (approximately 65%), which enhances their gelling capacity, and neutral sugar-containing polysaccharides (Ara in the rhamnogalacturonan type I—Rhamnogalacturonan (RG)-I) are responsible for their structural flexibility). Specific glycosidic bonds and backbone conformations form the primary structure of fruit polysaccharides [23,24].

Homogalacturonans (HG), the predominant pectin component, are made up of linear α-1,4-linked GalA chains of variable methyl esterification [25]. In citrus fruits, unesterified HG domains participate in “egg-box” complexes with Ca^2+^, thereby stabilizing the gelling matrix in situ. RG-I, a branched pectin type, contains alternating α-1,2-Rha and α-1,4-GalA residues in its backbone, substituted with side chains of Ara, Gal, or xylose (Xyl) [26]. RG-I increases the porosity of papaya and guava fruit cell walls, facilitating water uptake and fruit softening [27,28]. Heteropolysaccharides with other mixtures, including xylans and glucomannans, have mixed linkages (β-1,3/1,4 in hemicelluloses (HCs)) conferring mechanical strength [29]. Branched heteropolysaccharides in tropical fruits such as mango contribute to the preservation of bioactive compounds during ripening [30].

The molecular weight (MW) of fruit polysaccharides ranges between 1 kDa and more than 1000 kDa, and it directly affects solubility and function [31]. High-MW pectins (200–500 kDa), like those of passion fruit peels, form stable acidic gels, whereas low-MW fractions (<50 kDa) exhibit increased solubility and antioxidant activity [32]. Structural characteristics, such as branching and hydrophilic functionalities (hydroxyl and carboxyl groups), further influence solubility. For example, cellulose, with its crystalline β-1,4-glucan structure, is insoluble in water, unlike highly soluble β-arabinogalactans of kiwifruit, whose branched chains allow polar solvent interactions [33].

The composition of polysaccharides is more closely related to their action in fruit [34]. Cellulose, for example, has a structure that provides physical protection, with linear glycosidic bonds that display an insoluble characteristic, exhibiting no bioactivity in the human body upon ingestion [35,36]. Branched polysaccharides of low or medium molecular weight, which act as energy reserves and interconnections in food, are structured in a more branched manner, with greater availability of unlinked polar groups, thus presenting themselves as more soluble polymers and, consequently, better utilized by humans [37].

Post-biosynthetic modifications significantly change polysaccharide properties. Methyl esterification of the carboxylic acid groups in GalA in pectins influences gelling characteristics [38]. Strawberry pectins with greater than 70% esterification gel under low pH conditions [39], whereas banana pectins (~40% esterification) require Ca^2+^ for the network [40]. Acetylation, present in the xylans of pears, introduces hydrophobic groups, altering protein and lipid interactions and slowing enzymatic hydrolysis during storage [41]. In contrast, arabinan or galactan side chains in RG-I enhance water retention and toxin adsorption in the gastrointestinal tract, thereby promoting bioactivity [42,43]. Further, β-1,6-linked branches in dextrans regulate prebiotic and immunomodulatory activities when ingested by humans and fermented by gut microbiota [44]. Such modifications not only establish physicochemical characteristics but also broaden the applications of nutraceutical and bioactive encapsulation, underlining the contribution of fruit polysaccharides to human health [45,46].

These structural variations and post-biosynthetic adjustments not only shape the physical attributes of fruits, such as firmness, juiciness, and viscosity, but also determine their biological performance as functional foods. Highly branched Arabinogalactans exhibit strong immunomodulatory and antioxidant action, while de-esterified pectins contribute to cholesterol reduction and improved intestinal transit [45,46]. Thus, the structural diversity of polysaccharides represents an evolutionary adaptation to environmental stresses, directly connecting the specific conditions of biomes to the biochemical structure of fruits.

Despite advances in structural characterization, the relationships between the abiotic conditions of biomes and the compositional patterns of bioactive polysaccharides in fruits are still poorly explored. Temperature, humidity, radiation, and soil nutrients impose selective pressures that regulate gene expression and enzymatic activity in the biosynthetic pathways of these compounds. However, translational analyses that connect ecological parameters to molecular diversity remain poorly understood. Understanding these interconnections is essential to explaining how environmental gradients shape biochemical adaptations and determine the nutritional potential of fruits.

Thus, this narrative literature review aims to establish direct and indirect associations between the abiotic conditions that define the main terrestrial biomes and the polysaccharide composition of their native edible fruits. In addition, it aims to provide a global map of bioactive polysaccharides, highlighting their structural variability, functional implications, and ecological distribution. Finally, by quantifying the prevalence and diversity of polysaccharides across biomes, this review aims to define compositional limits and to offer a consolidated framework for future biochemical, nutraceutical, and ecological investigations of bioactive polysaccharides.

## 2. Methodology

This work is a narrative literature review that synthesizes structural, compositional, and ecological patterns of polysaccharides from edible fruits across global biomes. Literature searches were conducted in scientific indexers such as Web of Science, Scopus, PubMed, and Google Scholar using the following main combinations of keywords: “fruit polysaccharides”, “pectin”, “hemicellulose”, “monosaccharide composition”, “glycan structure”, “abiotic stress”, “native fruits”, and “biome”. Searches favored, but not restricted, publications from 2000 to 2025, and reference lists of retrieved papers were screened to recover additional relevant sources and classic references.

Biome delimitation and terminology followed the climatic and biogeographic framework proposed by Loidi et al. (2022) in Vegetation Classification and Survey [47], which served as the primary reference for defining the nine biomes analyzed.

For each biome, at least three native edible fruit species were selected for analysis. The qualitative species selection prioritized: (i) nativity to the corresponding biome; (ii) ecological or cultural integration within local food systems, including production data when available; and (iii) the existence of detailed biochemical or structural studies on fruit polysaccharides. When multiple studies existed for a given species, priority was given to those reporting comprehensive compositional, linkage, or extraction analyses.

Extracted variables included monosaccharide composition, degree of methylation or acetylation, dominant glycosidic linkages, molecular weight ranges, extraction methods, and reported bioactivities. The comparative synthesis emphasized recurring structural motifs, such as homogalacturonan, rhamnogalacturonan I and II, arabinogalactans, and xylans, and their functional implications under distinct abiotic stresses (drought, salinity, freezing).

## 3. Global Biomes and Native Edible Fruits: An Overview

A biome is defined as a large ecological community classified into climatic realms, which is differentiated by the physical structure of natural vegetation and mainly linked to specific climatic conditions [47].

It is characterized by substantial heterogeneity in abiotic conditions, which together determine its ecological character. In the case of Potential Natural Vegetation, excluding recent human-caused changes, nine terrestrial biomes are distinguished: (1) tundra; (2) boreal forest; (3) temperate deciduous forests; (4) temperate pluvial evergreen forests, shrublands and grasslands; (5) temperate aridiestival evergreen forests and shrublands; (6) steppe; (7) deserts and semi-deserts of arid regions; (8) tropical pluviseasonal forests and shrublands; and (9) tropical rainforests [47]. These biomes are found worldwide and can cover several continents, although they may have different species of vegetation, provided they share the same climatic conditions. The variety of ecological systems is expressed in their abiotic components, which include temperature, climate, vegetation characteristics, and soil type.

These variations operate to regulate the ecological processes of each system. Temperature, regulated by geographic characteristics such as latitude, elevation, and continental location, directly regulates metabolic processes and phenological cycles [48,49]. Extreme cold or heat imposes physiological limits, and organisms possessing thermal tolerance mechanisms, such as antifreeze or anti-stress molecules, are favored [50]. Climate, in terms of precipitation amount, distribution, and atmospheric humidity, regulates water availability, leading to adaptations such as deciduousness and deep rooting [51]. Soil characteristics, such as pH, organic matter content, and nutrient status, are influenced by pedogenic processes, including leaching, mineralization, and organic accumulation [52].

**Figure 2:**
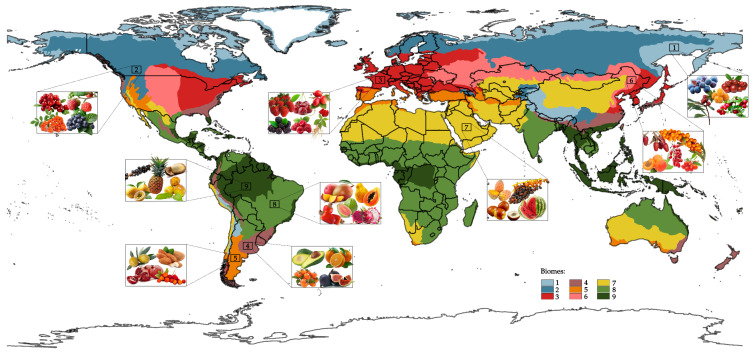
Geographical localization of each of the nine biomes and their native edible fruits analyzed. Drawings generated by the QGIS Bratislava v. 3.40.9 and Inkscape v. 1.4 software. All images are open source (Creative Commons).

The processes directly affect the health status of angiosperms, inducing mutualistic relationships that promote fruit development and are crucial to plant productivity and health [53,54]. Acidic and nutrient-deficient soils selectively favor plants with mycorrhizal relationships, whereas alkaline and dry soils selectively favor xerophytic or halophytic species [55]. These abiotic determinants form a hierarchical paradigm that defines species distribution and ecosystem processes. The relationship between ecological systems and their abiotic elements is responsible for the formation of edible fruits (Figure 2).

Seasonal climatic conditions enable the development of a diverse range of fruit species, which prosper due to specific changes that induce flowering and fruiting seasons [56]. Conversely, areas with low water availability have led to the development of fruit species with specialized water-holding capacity adaptations and tolerance to stressful environmental factors [57]. The unique abiotic factors in every ecosystem direct the evolutionary strategies of plant life, resulting in a diverse range of edible fruits that not only respond to the ecological conditions of their ecosystems but also exhibit a range of bioactive molecules characteristic of adaptations to their native ecological niches [58,59].

### 3.1. Biome of the Tundra: Cold-Adapted Fruits and Their Bioactive Polysaccharides

The tundra biome, which is found in Canada, Alaska (United States of America (USA)), Siberia (Russia), Greenland, Antarctica, and the Andes (South America), is characterized by permafrost (soil that remains permanently frozen) and experiences temperatures above freezing for only a few months, rarely exceeding 10 °C [47]. It has long, cold winters, with temperatures below −30 °C. Since the ground is perennially frozen, there is slow decomposition of organic matter, and the soil is composed of rocks, gravel, and sand, with an acidic pH, making it infertile. The growth of trees does not occur in low temperatures and specific soil types; only bushes, herbaceous vascular plants, bryophytes, and lichens will grow [47,60].

Edible fruits native to the tundra biome are adapted to harsh environmental and climatic conditions (Table 1). As dwarf shrub species dominate tundra vegetation communities, wild berries are the predominant fruit-bearing plants. At the same time, they have been extensively studied for their phenolic compounds, with a large body of significant contributions over many decades [61].

Despite this scarcity, some findings have been reported, such as the extraction and characterization of a new polysaccharide (MW: 4.98 × 10^4^ KDa) from the Arctic blueberry (*Vaccinium uliginosum*), designated as *Vaccinium uliginosum* polysaccharide (VUP-1). It is a heteropolysaccharide containing Ara, Man, GalA, Glc, and Gal, soluble in water and insoluble in ethanol, chloroform, and other organic solvents [62].

Lingonberry (*Vaccinium vitis-idaea*), less studied than other *Vaccinium* spp., has been of interest to researchers due to its phytochemical and nutritional composition [63]. The polysaccharide fraction from the hot water (90 °C) extraction of lingonberry press cake contained 36% total carbohydrates, 4.7% protein, and 3.5% phenolic compounds. Neutral monosaccharides (Glc, Ara, Gal) and acidic constituents (GalA, Glucuronic acid (GlcA)) were present, with traces of Rha, Fuc, Xyl, and ribose (Rib). Generally, the polysaccharides consisted of acidic polymers and polyphenol–polysaccharide conjugates, such as neutral arabinogalactans esterified with hydroxycinnamates [64].

Polysaccharide extraction of cranberry (*Vaccinium oxycoccos*) was carried out using high-pressure processing, and the conditions were optimized at 500 MPa, pH 13, and a 12-min pressure-holding time [65]. The process revealed the richness of the fruit in RG-I pectic polysaccharides, characterized by a high GalA content (46.31%) and a complex structure containing Rha and Ara residues; in contrast, RG-I accounted for 63.22% of the polysaccharide fraction. Structural monosaccharide composition analysis revealed a Rha/GalA ratio of 0.31, reflecting a homogeneous structure with a predominance of HG domains, and a ratio of 1.58 for (Gal + Ara)/Rha, reflecting enhanced branching of the RG-I backbone by arabinogalactan side chains. These results reflect a pectin structure with a predominance of methyl-esterified HG domains interspersed with branched RG-I regions, a structure found to be associated with cold-stress adaptation in tundra plants [66].

Cloudberry (*Rubus chamaemorus*), a nutritionally and culturally significant food in Latvia, is uncharacterized in terms of its polysaccharide composition [67]. Water ethanol (70%) extraction of cloudberry bioactive compounds proved the occurrence of free monosaccharides as the primary carbohydrate component, Glc (198 mg/g), Fru (47 mg/g), Xyl (8.4 mg/g), Gal (4.2 mg/g), and Ara (2.1 mg/g). Structural polysaccharides have not yet been reported [68].

Bearberry (*Arctostaphylos uva-ursi*), another tundra native fruit, lacks any published literature on the polysaccharide composition or monosaccharide profiling of its peel or pulp. However, the physicochemical characterization of its leaves is well-documented, indicating their potential ethnopharmacological applications [69].

### 3.2. Biome of the Boreal Forest: Berry Diversity in Northern Ecosystems

The taiga boreal forest biome, found in Canada, Siberia (Russia), Alaska, Scandinavia, and Hokkaido (Japan), experiences long, snowy winters with severe temperatures (around −40 °C) and brief, warm, wet summers with temperatures of 10–20 °C (Table 2). It experiences modest annual precipitation (500–1000 mm/year) [47]. The vegetation consists of coniferous forests and deciduous trees at the margins. They have podzolic, or acid, soils with an organic topsoil but without bare minerals. They drain quickly and have poor water-holding capacity, collectively restricting soil fertility [47,70].

Bilberry (*Vaccinium myrtillus*), typical of boreal biomes, exhibits cell wall polysaccharides shaped by its ecological specialization [71]. Sequential extraction of skin, pulp, and seeds revealed Glc-rich hemicelluloses (34 mol%) and cellulose (51 mol% in residue) as major components, while pectins occurred in lower amounts. Hot buffer- and chelating agent-soluble fractions were mainly homogalacturonan (HG; 83 mol% GalA) with moderate methylation (Degree of Methylation (DM) 60–77%) and little branching. Diluted alkali-soluble fractions contained rhamnogalacturonan I (RG-I) with short arabinan chains (Rha 3 mol%, Ara/Gal ≈ 1). Seeds were rich in Xyl (44 mol%), indicating highly cross-linked xylans (Degree of Acetylation (DA) > 100%) adapted for mechanical stiffness. After juice processing, the press cake retained 76% of native polysaccharides (39 mol% Xyl, 51 mol% Glc), consistent with cellulose–xylan networks. The juice fraction contained Ara (34 mol%), Gal (20 mol%), and GlcA (21 mol%) from enzyme-resistant RG-I and acidic hemicelluloses. Acetylation in alkali-soluble fractions mainly occurred on xylans, influencing solubility and enzymatic resistance [72].

Rowanberry (*Sorbus aucuparia*) pulp was found to contain water-soluble pectin with a high content of GalA (62–68%) and moderate DM (49–51%). Polysaccharides (total yield: 4.2% dry weight), obtained by sequential aqueous (68 °C) and ammonium oxalate (0.7%, pH 4.0) extractions, consisted of HG backbones intercalated by RG-I domains [73]. Neutral side chains are dominated by 1,5-linked Arabinose-furanose (Ara*f*) residues (SAII: 12.3% Ara) and branched 1,3,6-linked Galactopyranose/Mannose-pyranose (Gal*p*/Man*p*), with terminal Glucose-pyranose (Glc*p*) capping side chains, as indicated by methylation Gas Chromatography–Mass Spectrometry (GC/MS). Enzymatic hydrolysis with endo-polygalacturonase degraded 41–45% of pectins, producing resistant RG-I fragments rich in Ara (18.7–24%) and with high DM (70–73%), reflecting enzyme-resistant, branched regions. The polysaccharides displayed 37–53% trolox-equivalent antioxidant activity, which was likely mediated by arabinan–polyphenol interactions [73,74].

*Rubus chingii* is a raspberry species used in traditional Asian medicine to treat gastrointestinal diseases and inflammation [75]. A new acidic heteropolysaccharide (pRCP), obtained from young raspberry through optimized ultrasonic-assisted ethanol pretreatment followed by water extraction and ethanol precipitation (yield: 8.3%), has an MW of 74.86 kDa and satisfactory homogeneity [76]. Structural analysis indicated a backbone of →3,6)-β-D-Gal*p* and →5)-α-L-Ara*f* with side chains of α-Ara*f* (1→ residues attached at the C3 position of Gal. Monosaccharide composition was marked by Ara (39.76%) and Gal (39.43%), together with uronic acids (GalA: 8.56%; GlcA: 5.64%), Glc, Xyl, Man, and Fuc. Methylation and 2D Nuclear Magnetic Resonance (NMR) confirmed branched RG-I-like domains and terminal Glc*p* residues [76].

Cranberry (*Vaccinium macrocarpon*), native to boreal forests, develops structurally complex cell wall polysaccharides adapted to cold and nutrient-poor conditions [77]. Sequential extraction of alcohol-insoluble pomace fractions revealed predominance of pectic polysaccharides with clear compositional stratification. The hot buffer-soluble fraction contained methyl-esterified HG (75% DM; 72.8% GalA) with arabinan (10.5% Ara) and galactan (5.4% Gal) side chains as high-molecular-weight polymers (>10^2^ kDa) [78]. Chelating extraction yielded linear HG (83.4% GalA) with minimal branching and the highest yield (11% *w*/*w*). Diluted alkali fractions contained RG-I domains with Rha-rich backbones (1.6 mol%) and abundant arabinogalactan side chains [(Ara + Gal)/Rha = 11.5:1] and low DM (2%). Concentrated alkali fractions recovered hemicellulose glucomannans (30.2% Man/Xyl) and xyloglucans (35.9% Glc) with residual arabinan (12.9%) and galactan (13.8%). RG-I side chains consisted of α-1,5-Araf and β-1,4-Galp, while HG domains displayed variable methylation. These biome-specific polysaccharides range from gel-forming pectins to rigid hemicelluloses, reflecting adaptations for water retention, mechanical strength, and stress tolerance [78].

### 3.3. Biome of the Temperate Deciduous Forests: Seasonal Fruits and Polysaccharide Profiles

The temperate deciduous forest biome is located in regions such as France, Germany, Eastern China, Japan, the Eastern United States, and New Zealand (Table 3). Characterized by four seasons with cold winters and warm summers, this biome is distinctive. It experiences well-distributed rainfall throughout the year, ranging from moderate to high (600–1500 mm/year) [47]. The soil is chernozem: rich, organic, and humus, with a neutral pH, and has a varied herbaceous stratum. These characteristics are responsible for superior nutrient retention and agricultural yield, although speedy drainage and fluctuating mineral content can affect local fertility [47,79].

Strawberries (*Fragaria ananassa*) are the most widely commercialized berries found in the U.S.A. markets [80]. They have a complex polysaccharide composition, which differs according to cultivar, stage of ripeness, and harvesting conditions. The cell wall contains elevated levels of dietary fiber, predominantly pectins and HCs. Extraction of polysaccharides with boiling 70% ethanol and examination of the Alcohol-Insoluble Solids (AIS) reveals GalA as the predominant component (average 262 mg/g AIS), followed by cellulosic Glc (234 mg/g AIS). Neutral sugars are Xyl (77 mg/g AIS), Gal (76 mg/g AIS), and Ara (57 mg/g AIS), with Ara and Gal limited to pectin side chains. Pectins were highly methylated (DM: 60%) and structurally heterogeneous, with a linearity ratio (GalA/Rha) ranging from 7.1 to 36.1 (average 12.4), corresponding to HG-dominant regions [81]. RG-I is branched ((Gal + Ara)/Rha) between 4.3 and 10.0, while arabinogalactan side chains have an Ara/Gal ratio of 0.4–2.0 (0.9 average), corresponding to variability in side-chain substitution patterns among cultivars [81,82].

Polysaccharides extracted with hot water from sweet cherries (*Prunus avium*; cultivars Lapins, Skeena, Sweetheart), raspberries *(Rubus idaeus*) and American ginseng berries (*Panax quinquefolius*) contained 20–42% carbohydrate (cherries: 39.3–41.5%; ginseng pulp: 19.7%; raspberries: 28.9–34.8%), protein, and phenolic compounds [83]. The MW ranged from ~52 kDa for cherries, ~123 kDa for raspberries, and bimodal peaks (26 kDa, 13 kDa) for ginseng. Monosaccharide composition was highly variable, with a dominance of Ara, Gal, and Glc; raspberries had plenty of Ara; ginseng had plenty of Gal with minute quantities of Ara. Fourier Transform Infrared Spectroscopy (FT-IR) spectroscopy validated cherries’ uronic acids (peaks at 3200 cm^−1^ [–OH], 1700 cm^−1^ [C=O stretching], 1600 cm^−1^ [carboxylate]), raspberry-specific (1→4)-α-glucans (920 cm^−1^, 850 cm^−1^), and ginseng protein-conjugated polysaccharides (1500–1600 cm^−1^) with fewer uronic acid signals, indicating structural and functional distinction between species [84].

Ultrasound-assisted extraction of mulberry (*Morus nigra*) polysaccharides yielded 3.13% crude extract (MFP), further purified into MFP-1 (deproteinized) and MFP-2 (decolorized/deionized) with increasing carbohydrate purity (58.6–81.2%) and reduced protein content (16.5%→1.0%) [85]. Monosaccharide analysis showed that crude MFP was Glc-dominant (78.5%), whereas MFP-2 presented balanced Ara (36.0%), Gal (34.1%), and Glc (29.9%) proportions, with higher uronic acid (14.5%) than MFP (9.6%). FT-IR confirmed preserved structure, with bands for O–H (3400–3420 cm^−1^), C–H (2920–2935 cm^−1^), carboxyl (1613–1625 cm^−1^), and pyranose (1074–1078 cm^−1^) groups [86]. Increased uronic acid and reduced protein content correlated with stronger bioactivity: MFP-1 had higher antioxidant capacity (Oxygen Radical Absorbance Capacity (ORAC) 2808.6 μM TE/g), while MFP-2 showed the highest hypoglycemic effect via α-glucosidase inhibition (64% at 20 mg/mL) and reduced glucose diffusion. These results link compositional refinement—especially monosaccharide balance and uronic acid enrichment—to enhanced functional performance of mulberry polysaccharides [85,86].

Rose hip fruits (*Rosa canina*), rich in vitamin C, antioxidants, and bioactive compounds, have been traditionally used in both food and medicinal applications. One study purified its polysaccharides using citric acid (1%), which yielded a fraction characterized by a GalA-rich backbone (45.5%) complemented by neutral sugars, including Gal (5.5%) and Ara (4.7%) [87]. Structural analysis reveals an HG-dominant structure with alternating non-methylesterified domains and short RG-I segments, featuring high DM (62%) and DA (10%). The polysaccharide contains a heterogeneous MW profile (10–100 kDa) and a blockwise distribution of methyl and acetyl esters, which contribute to its structural complexity. New oligomers, such as unsaturated pentamers with dual methyl and acetyl substituents, demonstrate the presence of various functional groups. The structural characteristics indicate promising bioactive activities, including antioxidant and immunomodulatory effects, and the high DM favors applications demanding acid stability and resistance to gelation. Together, rose hip fruit pectin appears as a multifunctional biopolymer with specific characteristics, making it different from traditional pectin sources [87,88].

### 3.4. Biome of the Temperate Pluvial Evergreen Forest: Rainforest Gems and Their Functional Compounds

The temperate pluvial evergreen forest biome, occurring on the Pacific Coast (USA/Canada), Southern Chile, Tasmania, New Zealand, Japan, and Southeastern China, has moderate temperatures (5–15 °C in winter) [47] (Table 4). It is an ocean-influenced humid climate characterized by frequent precipitation, regular fog formation, and high annual rainfall (1000–3000 mm/year), primarily driven by orographic uplift. Soils are rich, usually volcanic (andisols) or organic (histosols), with high nutrient levels. Dominant vegetation is laurophyllous broadleaf evergreen trees, giant conifers such as Sequoia sempervirens in North America, and a dense understory of mosses, ferns, and epiphytes, indicative of hyperhumid temperate rainforest environments [47,89].

Avocado (*Persea americana*) is a climacteric fruit with unique physiological and biochemical characteristics, including postharvest ripening controlled by ethylene biosynthesis, the accumulation of monounsaturated fatty acids (primarily oleic acid) during mesocarp development, and the occurrence of seven-carbon (C7) sugars, which is unusual among other fruits [90,91]. Dry weight basis, the pulp contains approximately 60–70% lipids and 10% carbohydrates, 65–80% of which are dietary fiber made up of insoluble fractions (hemicellulose, cellulose) and soluble fractions (pectin). Significantly, the pulp of cultivar ‘Hass’ has high levels of the C7 monosaccharide mannoheptulose (9–347.6 mg/100 g fresh weight) and its reduced polyol counterpart, perseitol (16–424.2 mg/100 g fresh weight), which are produced through a distinct metabolic pathway not found in most angiosperms. These molecules, in addition to the fruit’s unusual lipid-based composition, highlight its nutritional and metabolic distinctiveness among plants [90,91].

Orange (*Citrus sinensis*) accumulates pectic polysaccharides in its albedo tissue, which has a high water-holding capacity and structural firmness. Fractionation through sequential extraction of AIS of albedo revealed pectins in the initial fractions and HCs in alkaline fractions. Sodium Acetate (NaOAc) and Chelating Agent (CDTA) solubilized methyl-esterified HG (83–85% GalA), the most significant amount being from CDTA (97 mol%). Sodium Carbonate (Na_2_CO_3_) extraction resulted in branched RG-I domains, rich in arabinan (31 mol% Ara) and galactan (33 mol% Gal) side chains, typical for structurally complex pectic matrices. Alkali extracts (1 M and 4 M Potassium Hydroxide (KOH)) were directed toward hemicellulose polymers, specifically xyloglucans (63–50 mol% Glc/Xyl), with the remaining arabinogalactans. RG-II, as defined by chromatography and enzymatic digestion, constituted 0.5% of AIS and contained characteristic sugars (2-O-methyl-Fuc/Xyl) and borate-cross-linked apiosyl residues, thereby confirming its conserved dimeric structure [92,93].

Persimmon (*Diospyros kaki*), a carotenoid- and polyvitamin-enriched fruit, is rich in free sugars (Glc, Fru) and structurally heterogeneous polysaccharides, as demonstrated by research on Uzbek cultivars (‘Khiyakuma’, ‘Tomapan’, ‘Zendzhi-Meru’). Stepwise extraction of Water-Soluble Polysaccharides (WSPs), Pectin Substances (PeSs), and HCs revealed that WSPs were the most prevalent fraction (3.7–7.6% yield), primarily comprising Glc, Gal, and Ara, with notable varietal and regional differences in monosaccharide ratios. PeSs (1.0–3.4% yield) contained GalA residues and were recovered by water-soluble polymers precipitated with aluminum sulfate. HCs, in contrast, showed biome-specific heterogeneity, with Glc being the dominant sugar in most cultivars, except in ‘Khiyakuma’ (Fergana), where the principal constituent was Ara. Structural analysis evidence indicates that WSPs contain Ara-rich side chains, whereas HCs are branched, suggesting potential differences in solubility and functional properties. Notably, cultivar ‘Zendzhi-Meru’ WSPs were rich in Glc (61.6%) and Gal (17%), whereas ‘Khiyakuma’ (Shirabad) ones contained HCs high in Ara (37.5%). The persimmon’s polysaccharides are regionally adapted biomolecules with compositional plasticity, rendering them promising candidates for nutraceutical applications, particularly in dietary fiber-enriched functional foods [94,95].

Fig fruit (*Ficus carica*) fruit polysaccharides (FPs) exhibit a heterogeneous composition comprising Glc, Gal, Ara, and Rha, with geographical and varietal variations in the proportions of these monosaccharides [96,97]. Sequential extraction steps reveal WSPs as the predominant fraction (3.7–7.6% yield), followed by PeSs (1.0–3.4%) and HCs (1.6–3.8%). Structural characterizations distinguish branched heteropolysaccharides with α-1,4 and β-1,3,6 glycosidic bonds, such as the HG domains of Pectins and xyloglucans in HCs. The MW range varies from 21.9 to 6890 kDa, depending on the extraction techniques. Subcritical water extraction yields high-molecular-weight polymers (56.48% yield), while enzymatic treatments produce lower-weight fragments (34.13% yield). Biome-specific adaptations are evident, with high Gal content in tropical cultivars (‘Zendzhi-Meru’ WSPs: 61.6% Glu, 17% Gal) and Ara-bearing HCs in the ‘Khiyakuma’ cultivar (Fergana, 37.5% Ara). Side-chain substitutions are confirmed by high-performance characterization (High-Performance Anion-Exchange Chromatography with Pulsed Amperometric Detection (HPAEC-PAD), GC-MS), i.e., arabinogalactans and RG-I. In contrast, uronic acids constitute up to 53.5% of PeSs. These structural views highlight the compositional heterogeneity and environmental responsiveness of FPs as biochemically distinct polymers, making them worthy of further research in material and food-related applications [97].

### 3.5. Biome of the Temperate Aridiestival Evergreen Forests: Mediterranean Flora and Polysaccharide Potential

Found in Spain, Italy, California (USA), Central Chile, the Cape Region (South Africa), and Southwestern Australia, the Aridiestival Evergreen Forest Biome matches the temperate aridiestival evergreen forests with summer aridity, more than two consecutive months without water and low to moderate yearly precipitation (300–800 mm/year), all in winter [47] (Table 5). Soils are generally shallow, stony, and poor in nutrients, and they are classified as lithosols or Terra Rossa (Mediterranean-type soils), characterized by low organic matter and high mineral leaching. Vegetation is dominated by sclerophyllous shrubs with pyrophytic adaptations (fire-resistant features), tough, waxy leaves to reduce water loss, and deep roots to access groundwater. Drought-deciduous plants and geophytes adapted to seasonal water stress occur in specific locations [47,98].

Olive fruit (*Olea europaea*), a Mediterranean biome drupe, undergoes extensive cell wall modification during ripening, reflecting biome-specific abiotic stresses such as temperature fluctuation and water availability [99]. Sequential extraction of cell wall material revealed a rising ratio of hot buffer-soluble pectins, from 40% to 60% of the total extractable pectin, indicating enhanced pectin solubilization throughout ripening. The HG and RG-I domains showed decreased DM (68% to 59%) and DA (72% to 63%) in purple-stage fruits, which correlated with reduced polysaccharide cross-linking. Neutral sugar profiling revealed a 30% decrease in Ara content and increased Rha-to-uronic acid ratios, indicating increased RG-I branching in ripe fruits. Hemicellulose fractions extracted with 1 M and 4 M KOH were compositionally stable during ripening phases, with xyloglucans (Xyl/Glc ratio: 0.6–0.75) and acidic xylans (4-O-methyl-GlcA substitutions) as predominant components. MW profiles of pectic and hemicellulose polymers indicated minimal depolymerization, highlighting structural preservation despite solubilization modifications [100].

Almond (*Prunus amygdalus*), a dense fruit, synthesizes structurally variable polysaccharides in its edible tissues, a manifestation of adaptive strategies to dry climates [101]. Sequential extraction of pectic polysaccharides in the fruit’s outer peels showed HG and RG-I domains, with hydrochloric acid-soluble fractions having the highest content of GalA (36.1%) and low DM (31.76%). These polymers from peel, which are made up of Ara (33–39%), Gal (49–57%), and Man (2–10%), are of low MW (108 kDa) and high uronic acid density. In the seed cotyledons, analysis of cell wall material revealed the presence of Ara-rich pectic polysaccharides (45 mol%) in addition to xyloglucans and acidic xylans, in a cellulose-hemicellulose matrix. Alkaline extraction (4 M KOH + H_3_BO_3_) solubilized a gel-forming arabinan-pectic fraction (55 mol% Ara, 23 mol% uronic acids), which is spatially localized in the microfibril network of the cell wall, indicating a structural reinforcement role. Seed xyloglucans, defined by Glc-Xyl ratios (4:3) and arabinan side chains, were enriched in alkali-soluble hemicellulose fractions [101,102,103].

Pomegranate (*Punica granatum*), a fruit native to arid regions of Afghanistan and Iran and grown globally, accumulates structurally diverse polysaccharides in its edible tissues [104]. The peel, one of the richest sources, contains pectin with HG domains as the predominant constituent, GalA constituting 46–68% of uronic acids, and MW ranging from 422 kDa to 18,631 kDa depending on extraction methods. These pectins have high DM (5.1–74.0%) and DA (up to 18.6%), particularly in ‘Purple Queen’ cultivars, where F3 fractions have DM 74.0% and DA 18.6%. Structural studies describe branched arabinans with (1→5)-linked α-L-Ara*f* backbones and (1→6)-β-D-Gal*p* side chains in ‘Wonderful’ varieties with minor RG-I residues. ‘Purple Queen’ peels show typical Glc-rich glucans (23.8 mol%) and reduced arabinan branching compared to ‘Wonderful’ cultivars. Seeds yield galactomannans, containing a β-1,3-linked Gal backbone with β-D-Man side chains. Arabinogalactans and glucans (6–8% yield) are produced by leaves and flowers, with (1→3,6)-linked galactan and (1→4)-glucan structures. Uronic acid content varies by variety, with ‘Wonderful’ cultivars containing more (50–63%) and ‘Purple Queen’ (49.9%) having the lowest [104,105,106].

*Arbutus unedo*, a Mediterranean shrub or tree producing small, spherical fruits with a gritty pulp, contains a cell wall polysaccharide composition of Glc (22.19–23.95%), Xyl (16.54–18.84%), and Ara (~2.65%) as the predominant neutral sugars in AIS [107,108]. The neutral sugars indicate a structural framework rich in cellulose and HCs, particularly xylans and xyloglucans, as evidenced by high Xyl-to-Glc ratios (~0.7). FT-IR revealed dynamic remodeling of pectin throughout ripening, with green fruits having high methyl-esterified HG domains that declined in the red stages as de-esterification increased, which correlated with reduced firmness. RG-I pectic domains were detected, with Ara and Gal side chains pointing to branched structures. Scanning electron microscopy highlighted tightly bunched scleroids with secondary cell wall thickening, in agreement with lignin content of up to 25.68% in ripe fruits. Linked with Xyl-rich HCs, this lignified matrix underscores a rigid cell wall architecture adapted to Mediterranean drought conditions, balancing structural integrity with polysaccharide solubilization during ripening [107,108].

### 3.6. Biome of the Steppe: Hardy Fruits of Semi-Arid Landscapes

The steppe biome is characterized by extreme continentality, with an annual temperature range exceeding 40 °C, cold winters (often below −20 °C), and warm summers (reaching temperatures as high as 30–40 °C) [47] (Table 6). In the semiarid interiors of the Great Plains (USA), Eurasian Steppe (Ukraine, Kazakhstan), and Patagonian Plateau (Argentina), it has low, unpredictable rainfall (250–500 mm/year), predominantly in spring/summer, resulting in grass dominance by perennials and drought-tolerant shrubs with xerophytic adaptations, i.e., deep taproots and low leaf area. Soils are predominantly mollisols (chernozem in Eurasia and North America) with thick horizons rich in organic material, high base saturation, and calcareous subsoil horizons. More aridic aridisols and lower fertility characterize Patagonian steppes [47,109].

Sea buckthorn (*Hippophae rhamnoides*), a deciduous shrub from high-altitude Asian and European regions, adapts to harsh environments with extreme temperature shifts and poor soils [110]. Polysaccharides extracted from its berries by hot water, ultrasonic, microwave, or ethanol-assisted methods show high structural diversity, with over 20 fractions (4.9–6260 kDa) isolated. Monosaccharide analysis revealed heteropolymers rich in GalA, Glc, Gal, and Ara, with minor Rha, Xyl, and Man. Structural characterization identified HG-type pectins with α-(1,4)-GalA backbones, branched heteroglycans containing →4)-β-D-Glcp-(1→ and →3,4)-β-L- Rhamnose-pyranose (Rha*p*)-(1→ residues, and arabinogalactan side chains composed of α-L-Araf and β-D-Galp [110]. Similarly, hot water–extracted WSPs from *Berberis vulgaris* (inflorescences, fruits, leaves) exhibit pectic heteropolymers with high uronic content (82.3–86.8%) and GalA-based backbones [111]. Monosaccharide profiles include D-GalA, D-Gal, D-Glc, L-Ara, D-Xyl, and L-Rha, with Ara and Gal prevailing (ratios 5–6:4) and Rha > Xyl in inflorescences. These branched pectin-like structures, combining HG domains and arabinogalactan chains, reflect biome-specific adaptations to thermal stress and low nutrient availability [111].

Goji (*Lycium barbarum*), a nutrient-rich fruit, contains structurally heterogeneous polysaccharides with a predominance of pectic components [112]. A study employed various polysaccharide extraction methods, which, by alkaline extraction at low temperatures (32 °C), gave RG-I-enriched fractions containing long arabinan and galactan side chains, as shown by high (Ara + Gal)/Rha ratios (7.77) and Rha/GalA ratios (0.527), typical for branched structures. In contrast, high-temperature acid extraction (85 °C, 0.4% Hydrochloric Acid (HCl)) isolates HG-enriched polysaccharides with simpler side-chain complexity and reduced arabinan loss, as reflected in decreased (Ara + Gal)/Rha ratios (4.9–5.82) [113]. Polysaccharides extracted with hot water yield disordered, acidic heteropolymers with no predominance of RG-I or HG. Molecular analyses reveal that alkali-derived polysaccharides have significantly higher MW (7162 kDa) and branched morphologies by Size Exclusion Chromatography coupled with Multi-Angle Light Scattering (SEC-MALLS) and Atomic Force Microscopy (AFM). Comparison with acid-extracted fractions shows lower MW (199–717 kDa) and stiff linear conformations. Spectroscopic characterization confirms the presence of β-glycosidic linkages in the polysaccharides, with alkaline treatment resulting in complete de-esterification, as indicated by the absence of peaks at 1740 cm^−1^ (FT-IR) and non-esterified carboxyl groups in the NMR spectra. Sequential extraction reveals that mild alkali, following acid treatment, retrieves residual RG-I domains, indicating solvent-dependent release of structurally distinct pectin fractions [113].

Apricots (*Prunus armeniaca*) contain typical cell wall polysaccharide compositions with pectic components prevailing, and Ara (36.7–47.3%) and Gal (19.6–22.8%) as predominant neutral sugars in their AIR, which is consistent with RG-I-rich pectins having arabinan and galactan side chains. *P. armeniaca* gum exudate contains this arabinogalactan structure with 41.5% Ara and 23.7% Gal, along with 6–12% uronic acids for polyelectrolyte characteristics and enhanced solubility. Acid-soluble and alkaline/Ethylenediaminetetraacetic Acid (EDTA)-soluble pectin fractions highlight the dominance of Ara (57–70%) and Gal (14–22%) by the branched RG-I domains. Hemicellulose fractions, though Glc-predominant (31–37%), retain Ara (3–7%) and Man (10–12%) proportions, distinguishing apricots from peaches, which contain less Man and more Ara in hemicellulose. The higher Man level and Gal/Glc/Xyl-to-Man ratio (twice that of peaches) of the gum exudate can explain its functional versatility as an emulsifier and stabilizer, assisted by its protein-polysaccharide conjugates (2–3% protein) and high MW (~5.69 × 10^5^ g/mol). Unbiased sugar ratios (Ara/Glc) and structural fingerprints (arabinogalactan-proteins) provide robust biomarkers for authenticity verification in complex matrices, leveraging apricot-specific adaptations to biotic-mediated abiotic stresses [114].

Fruits of *Nitraria retusa*, adapted to saline-alkali and arid environments, yield polysaccharides with varied structural and bioactive character [115]. Response surface methodology-optimized ultrasonic-assisted extraction yielded three fractions (NRFP-1 (20.01 kDa), NRFP-2 (28.96 kDa), and NRFP-3 (67.45 kDa)), mainly composed of Glc (6.10–47.22%), Gal (15.97–43.07%), and Ara (21.28–46.33%), with uronic acids (3.40–9.26%) conferring polyelectrolyte nature. Structural analysis revealed heteropolysaccharides with α- and β-glycosidic linkages, α-(1→6) galactan backbones and branched arabinan side chains, supported by FT-IR absorbances at 868 cm^−1^ (α-configuration) and 763 cm^−1^ (β-pyranose). NRFP-3, which contained Ara (46.33%) and Man (6.96%), had higher antioxidant activity, scavenging 37.13% DPPH, 75.50% ABTS, and 45.90% hydroxyl radicals at 1.0 mg/mL, due to its higher MW and uronic acid content (9.26%), which improves solubility and electron donation. Comparative studies indicate regional diversity: Tunisian *Nitraria retusa* polysaccharides contained higher Glc (41.4%) and GalA (30.5%) contents, whereas Chinese variants contained arabinogalactan structures. Protein-polysaccharide conjugates (8.69%) and zeta potential scores (−9.27 to −19.8 mV) corroborate emulsification capacity and colloidal stability [116].

### 3.7. Biome of the Deserts and Semi-Deserts: Drought-Resilient Species and Bioactive Components

Situated in dry areas such as the Sahara Desert (Africa), the Gobi Desert (Mongolia/China), the Sonoran Desert (USA/Mexico), the Atacama Desert (Chile), and Australia, the desert and semi-desert biome is characterized by intense aridity, with hyperarid to semiarid climates [47] (Table 7). The yearly precipitation is very low (<250 mm/year), irregular, and spatially inconsistent, with significant diurnal temperature variations (>30 °C). Due to domination by sandy, saline, or rocky soils (Aridisols or Entisols), vegetation cover is sparse (<10% of total cover). It is composed mainly of drought-adapted vegetation, such as cacti, succulents, and xerophytic shrubs, featuring characteristics like Crassulacean Acid Metabolism (CAM) photosynthesis, reduced leaf surface areas, and extensive root systems for water collection [47,117].

*Opuntia ficus-indica* (prickly pear) is a xerophytic cactus that yields mucilaginous cladodes and fruits and is highly adapted to semiarid and arid biomes [118]. The polysaccharide component of its cladodes contained two structurally distinct high-MW components. One was a linear β-(1→4)-galactan with a homopolymeric backbone of β-D-Gal*p* units, as established by methylation analysis that yielded 4-linked Gal units and NMR spectroscopy that indicated anomeric signals and C-4 downfield shifts. The second fraction was a branched complex xyloarabinan with an α-(1→5)-linked Ara*f* backbone, substituted at positions *O*-2 and *O*-3. Branching occurred through terminal α-Ara*f* residues and new β-Xylose-pyranose (Xyl*p*) capping units, with Xyl substitution established by HMBC correlations between Xyl H-1 and Ara C-5. Methylation analysis also indicated 2,5-; 3,5-; and 2,3,5-linked Ara*f* residues, with a complex branching pattern. The arabinan core was also formed by 5-linked Ara*f* chains, with side chains terminated by Xyl units, a new structural aspect not previously reported for *Opuntia* polysaccharides. Galactan and xyloarabinan were present as separate entities, as corroborated by the absence of inter-residual Nuclear Overhauser Effect (NOE) contacts in the NMR spectra, though physical separation was not achievable [119].

Jujube (*Ziziphus jujuba*), a fruit native to arid and semiarid biomes, contains polysaccharides characterized by structural complexity and diversity [120]. Extraction methods, including hot water (ZJP-2b, isolated from *Z. jujuba* cv. ‘Hupingzao’), enzyme-assisted, and subcritical water techniques (LZJP3, derived from *Z. jujuba* cv. ‘Linzexiaozao’), yield polysaccharides with MW ranging from 6.5 kDa to over 2000 kDa. Structural analyses reveal two predominant types: RG-I pectic polysaccharides and branched arabinans. RG-I structures, exemplified by ZJP-2b (89.2 kDa), feature backbones of α-(1→4)-linked GalA interspersed with α-(1→2)-linked Rha residues, substituted at *O*-4 of Rha by side chains of β-(1→4)-galactan and α-(1→5)-arabinan. Branched arabinans, such as LZJP3 (97.7 kDa, extracted via subcritical water), exhibit α-(1→5)-linked Ara*f* cores with substitutions at *O*-2/*O*-3 by terminal α-Ara*f* or β-Gal*p* units. Monosaccharide composition is heterogeneous: ZJP-2b is composed of Rha (32.5%), Ara (5.9%), and Gal (7.9%), whereas LZJP3 is Gal and alduronic acid-rich (Gal:AldA = 2.05:6.84). Excessive GalA (46–74.7%) of these polysaccharides is present in methyl-esterified HG domains necessary for arid adaptation hydration. Analyses of linkages reveal 1,3,5-; 1,2,4-; and 1,4,6-branching schemes, with or without sulfation or carboxymethylation modifying solubility. Structural heterogeneity, such as branching density ((Gal + Ara)/Rha ratios as high as 1.58), highlights their biome-specific functional adaptability [121].

Watermelon (*Citrullus lanatus*), a widely cultivated fruit that grows in hot, dry, and semiarid climates, produces biomass with structurally complex polysaccharides [122,123]. Papain-assisted enzymatic extraction yielded a water-soluble heteropolysaccharide (PWR) comprising Gal (38.26%), Ara (26.12%), Rha (17.86%), Man (9.94%), Xyl (5.10%), and Glc (2.70%), with a 45% uronic acid content. NMR and SEC/MALLS-based structural elucidation indicated a pectin-like framework with arabinogalactan attached to RG-I. The arabinogalactan backbone consisted of β-(1→6)-D-Gal*p* units branched at *O*-3 by α-L-Ara*f* residues, while RG-I segments featured →4)-α-D-Gal*p*A6Me-(1→ repeats interspersed with α-L-Rha*p*-(1→4)-α-D-Gal*p*A6Me-(1→ motifs. Side chains included short Ara (α-1,5-linked) and Gal (β-1,3-linked) branches, alongside minor α-D-Man*p*-3-OAc substituents. SEC analysis indicated a weight-average molar mass of 484,000 g/mol with a compact, highly branched conformation (Mark–Houwink exponent: 0.43), consistent with adaptations to water retention in drought-prone environments [124].

Date Palm (*Phoenix dactylifera*), a keystone species of dry and semi-dry biomes [125], produces fruits containing differentiated polysaccharide-rich tissues: the lignified seed and the fleshy mesocarp. The mesocarp comprises ~14% polysaccharides, primarily featuring a unique (1→3)-β-D-Glc*p* backbone with (1→6)-branched structures, as well as arabinoxylans and pectic substances. In contrast, the seed comprises ~80% dietary fiber and is rich in hemicellulose xylan, featuring a β-(1→4)-D-Xyl*p* backbone substituted with α-L-Ara*f*, D-Gal*p*, and acetyl side chains. Structural analyses reveal seed-specific galactomannans, including a (1→4)-β-D-Man*p* core branched at *O*-6 by single α-D-Gal*p* residues, with Man-to-Gal ratios of 2.7:1. Alkali-soluble heteroxylans in seeds further display 4-*O*-methyl-α-D-GlcA substitutions at *O*-2 of Xyl residues. SEC analyses reveal that mesocarp glucans possess an MW equivalent to compact, highly branched conformations. At the same time, seed xylans are hydrophilic and structurally heterogeneous, indicating adaptation for water retention and mechanical strength under desert conditions [126].

### 3.8. Biome of the Tropical Pluviseasonal Forests: Seasonal Tropical Fruits and Their Polysaccharides

The biome of tropical pluviseasonal forests, such as the Cerrado and Amazonian savanna regions (Brazil), Gran Chaco (Paraguay/Argentina), East African savannas (Tanzania/Kenya), Indian monsoon forests, and Northeastern Australian tropical forests, is characterized by a pronounced pluvioseasonal climate [47] (Table 8). The biome features a distinct wet and 3–6 months dry season, with an annual rainfall of 1000 to 2000 mm. The vegetation is suited to seasonal water stress, with semi-deciduous trees with thick, fire-resistant trunks and deep taproots. The mean temperatures are high (25–30 °C) throughout the year. Soils are mainly oxisols or ultisols (highly weathered) in wet areas and alfisols (moderately fertile) in dry areas, usually clay-rich, lateritic, acid, and poor in nutrients as a result of long-term leaching and aluminum toxicity [47,127].

Papaya fruit (*Carica papaya*), which is a tropical climacteric fruit, produces water-soluble pectin-rich fractions (WSFs) with differing ripe and unripe structural profiles [128]. Extraction of WSFs indicated that ripe papaya (32%) yielded higher amounts than unripe papaya (23%), and both were composed mainly of pectin polymers. Monosaccharide analysis of composition showed GalA to be the predominant constituent (69–74% mol), followed by Glc (7.5–19.9% mol) and Gal (4.2–10.1% mol), ripe pectin containing less GalA, Ara, and Gal but more Glc. Structural characterization indicated an HG-dominant backbone, with unripe pectin containing longer RG-I side chains (Gal + Ara/Rha = 12.83 vs. 5.62 ripe) and higher MW polysaccharides. Both showed DM~45%, whereas NMR corroborated acetyl groups on GalA residues and a lowered RG-I backbone ratio (RG-I/HG) in ripe pectin by the action of polygalacturonase during ripening. Enzymatic breakdown associated with ripening also depolymerized β-1,4-glucans from cellulose to add to the Glc content. Such structural changes point toward the dynamic reorganization of the pectin structure during papaya ripening [129].

Mango (*Mangifera indica*) undergoes significant pectin remodeling during ripening, characterized by distinct arabinogalactan and RG fractions [130]. Arabinogalactan (Fraction I) comprises a 1→4-β-D-galactopyranan backbone branched at O-3 by 1→5-α-L-Ara*f* side chains, with a Gal-to-Ara ratio of 3:1. RGs (Fractions II and III) are dominated by 1→4-α-D-GalA residues (69% and 62% uronic acid, respectively), interspersed with 1→2-linked α-L-Rha in the main chain. Neutral side chains in these fractions are terminal Ara*f* residues and 1→6-linked β-D-Gal*p* branches, with minor 1→3,6-linked Gal branch points. Methylation, FTIR, and ^13^C NMR structural analyses established α-anomeric configurations and esterified carboxyl groups. Extensive depolymerization during ripening decreases the MW of these fractions significantly, Fraction II from 1300 kDa (unripe) to 21 kDa (ripe), concomitant with an 85–93% decrease in abundance. This breakdown of pectic architecture is linked to the solubilization of cell wall matrices, resulting in textural softening due to the loss of structural cohesion [130,131].

Guava (*Psidium guajava*) is cultivated in warm, humid environments with well-drained soil [132]. Polysaccharides have been extracted from guava fruits and leaves using hot water, microwave-assisted extraction, and ultrasonic-assisted extraction, resulting in heteropolysaccharides with diverse structural profiles. One of the dominant polysaccharides obtained from fruits had a backbone of (1→5)-α-L-Ara, (1→2,3,5)-α-L-Ara, and (1→3)-α-L-Ara residues, and Glc and Ara were present in a molar ratio of 9.92:84.06 with a MW of 1.6 × 10^4^ Da. One fraction contained a branched structure with (1→3)-linked α-L-Ara and (1→3,6)-linked β-D-Gal units, whereas Heteropolysaccharide-I (PS-I), a fruit heteropolysaccharide, consisted of 2-O-methyl-L-Ara, 2-O-acetyl-D-Gal, and D-methyl galacturonate in the ratio 1:1:1. Alkali extraction of a heteropolysaccharide yielded a Gal content of 31.29% with a complicated branching, consisting of Rha (6.98%) and GlcA (6.03%) residues. Such structural differences, as guided by protocols of extraction and plant tissue sources, demonstrate the compositional versatility of guava polysaccharides, indicating their functionality in cell wall structure and stress tolerance in tropical environments [133].

Cashew (*Anacardium occidentale*) produces an exudate polysaccharide characterized by a branched heteropolysaccharide containing a β-D-Gal*p* skeleton (72%) as the backbone structure. The minor components are α-D-Glc*p* (14%), α-L-Ara*f* (4.6%), α-L-Rha*p* (3.2%), and β-D-GlcA (4.5%), with no 4-O-methylGlcA, distinguishing it from other local species [134]. Structural determination by ^13^C NMR revealed a highly branched galactan core with three linkage patterns, 1→3, 1→6, and 1→3,6, resulting in a complex structure. The backbone is composed of β-D-Gal units, and side chains are terminated by GlcA residues, which confer the polymer its acidic character. Ara and Rha units are peripheral, most probably as terminal decorations or short side chains. MW distribution measurements by gel permeation chromatography showed two major polysaccharide groups at 28,000 and 67,000 g/mol, with minor high-molar-mass fractions (>500,000 g/mol) from polysaccharide-protein complexes. Branching density, as represented by the 6-O-substituted Gal units (30% of total Gal residues), and the prevalence of uronic acids represent hydration and structural strength adaptations to the tree’s native-dry habitats [134].

The dragon fruit (*Hylocereus undatus*), a tropical cactus plant native to dry and semi-dry environments [135], contains a water-soluble polysaccharide (DFPP), which is a complex heteropolysaccharide in nature. DFPP is composed of a molecular weight of 2.2 × 10^3^ kDa and Ara (2.3), Gal (3.4), GlcA (2.8), and Rha (1.0) in some molar ratios. Structural characterization, performed using ^13^C NMR and GC-MS analyses, indicated a branched backbone with predominantly 1→4-β-D-GlcA (Glucuronic-pyranose Acid (Glc*p*A)), 1→6-β-D-Gal (Gal*p*), and 1→4-α-L-Rha (Rha*p*) units. The backbone structure alternates between Glc*p*A and Gal*p* units, which are attached through 1→4 and 1→6 bonds, with Rha*p* providing structural rigidity via 1→4 bonds. A unique side chain extends from the backbone made up of α-L-Ara*f* residues in the arrangement α-L-Ara*f*-(1→5)-α-L-Ara*f*-1→, and is linked to →3,6-β-D-Gal*p* branching points. This forms a highly substituted galactan core with GlcA contributing acidic nature and Ara contributing terminal adornments. The unique inclusion of Rha*p* and Glc*p*A in the structure distinguishes DFPP from typical arabinogalactans, with structural adjustments that are appropriate to the arid climate of the fruit. Such structural complexity has a deep relation with DFPP’s role in maintaining cell wall integrity and aiding in water retention within *Hylocereus* spp. [136].

### 3.9. Biome of the Tropical Rainforests: Biodiversity Hotspots for Bioactive Compounds

The Tropical rainforest biome, including the Amazon Basin (Brazil, Peru, Colombia), the Congo Basin (Central Africa), and the Southeast Asian archipelagos (Indonesia, Malaysia), is characterized by receiving more than 2000 mm/year of high annual rainfall, with no distinct dry season (Table 9). The environment in these biomes has a uniformly hot and humid climate, with temperatures staying stable (25–28 °C) throughout the year. Biodiversity abounds in this biome, characterized by a multi-strata canopy comprising emergent, canopy, understory, and forest floor strata [47].

The soils are essentially oxisols and ultisols, which are highly weathered, clay, and leached of nutrients through heavy precipitation. There is high microbial activity in the form of rapid decomposition that propels a rapid nutrient cycle where organic material is rapidly cycled into biomass and not conserved in the soil. This results in low natural fertility despite dense cover [47,137].

Açaí (*Euterpe oleracea*), a palm fruit native to the tropical rainforest biome of the Amazon Basin, is noted for its purple skin and lipid-rich mesocarp [138]. Aqueous extraction followed by ethanol precipitation of the polysaccharides from açaí pulp yielded three predominant fractions (Acai-1, Acai-2, Acai-3) after Diethylaminoethyl cellulose (DEAE cellulose) chromatography. Acai-1, the largest polysaccharide fraction (∼200 kDa), was composed of Ara (47.0%), GalA (28.4%), and Gal (11.5%), with minor components of Rha (4.5%) and GlcA (3.0%) [139]. Structural characterizations indicated type II arabinogalactans with β-(1,3)-galactan as the backbone substituted with Ara and Gal branches, along with HG domains indicated by high contents of GalA. Acai-2 (26–60 kDa) and Acai-3 (4–12 kDa) exhibited similar monosaccharide profiles, but with increased Glc (10.4–18.8%) and decreased Ara (18.8–26.2%), indicating molecular heterogeneity. NMR spectroscopy detected methyl esters and acetyl groups, indicating partial esterification of GalA residues, which is consistent with the structural flexibility. Yariv reagent assays confirmed the presence of arabinogalactan in all fractions. Fluorescence spectra detected trace aromatic conjugates from residual polyphenol-polysaccharide interactions, indicating the complexity of açaí’s polysaccharides, which are defined by arabinogalactans and pectic substructures [138,139,140].

Pineapple (*Ananas comosus*), a tropical fruit of sweet-sour flavor and fibrous pulp, contains water-soluble polysaccharides with unique structural profiles [141]. Three fractions (Pineapple Polysaccharides (PAPs) 1–3) were isolated by hot water extraction, ethanol precipitation, and DEAE chromatography. PAP-1 (4.1 kDa) was composed of Ara, Xyl, Man, Glc, and Gal with a molar ratio of 1.44:1:16.05:5.05:4.11, while PAP-2 (1910 kDa) was composed of Rha and high GalA content (9.94% *w*/*w*). PAP-3 (2320 kDa) had no Glc but Xyl as the predominant monosaccharide (4.29 mol%). Structural analysis revealed PAP-2′s backbone as →4)-α-D-Man*p*-(1→2,4)-α-D-Man*p*-(1→, with branching at O-4 of Man units. Ara units in α-L-Ara*f*-(1→ and →3)-α-L-Ara*f*-(1→ linkages, as well as →4)-β-D-Gal*p*-(1→ and methyl-esterified →4)-α-D-Gal*p*AMe-(1→ linkages, contributed to heteropolysaccharide complexity. NMR spectra confirmed partial esterification of GalA in all fractions, with the highest level of methylation in PAP-2. These structural results reveal the complexity of pineapple’s polysaccharides, which feature man-rich backbones, arabinogalactan-like branched motifs, and diverse uronic acid modifications [142].

Cupuaçu (*Theobroma grandiflorum*), a tropical fruit native to the Brazilian Amazon, is characterized by its aromatic, creamy pulp and economic value in local food industries [143]. Polysaccharides obtained from cupuaçu pulp by aqueous extraction (25 °C) gave a major pectic fraction (W-1, 7% yield) that consisted of 65 mol% GalA with high methyl esterification (DE 53%) and low acetylation (DA 1.7%). Structural analysis revealed a heteropolysaccharide with HG region-dominant composition with alternating RG-I segments. The RG-I backbone was constructed from →4)-α-D-Gal*p*A-(1→ and →2,4)-α-L-Rha*p*-(1→ linkages, and the latter one was *O*-4-substituted at Rha with Gal-rich (13 mol%) and Ara-rich (6 mol%) side chains; minor components included Xyl (4 mol%), Man (3 mol%), and trace Glc, reflecting residual starch. NMR spectroscopy corroborated the presence of esterified GalA units (δ 100.1 ppm) and non-esterified residues (δ 99.3 ppm), together with signals assigned to arabinogalactan side chains (δ 109.1 ppm for α-L-Ara*f*). Methylation analysis also revealed the prevalence of 4-O-substituted GalA (78.2%) and 2,4-di-O-substituted Rha, indicating a pectin structure with smooth HG domains and branched RG-I regions [144].

Passion fruit (*Passiflora edulis*), a tropical fruit native to Brazil, is distinguished by its intensely aromatic pulp with a sweet and sour flavor [145]. Its peel polysaccharide fraction (PFCM), soluble in water and obtained through hot water extraction (90–100 °C, pH 6.0), revealed a heteropolysaccharide comprising a backbone of (1→4)-linked esterified and unesterified GalA residues, and neutral sugars Ara, Rha, Glc, Man, and Fuc, with minor amounts of Xyl and Rib. Structural analysis demonstrated a pectin-like HG and RG-I domain structure, confirmed by a low GalA/Rha ratio (due to RG-I prevalence) and NMRs for methyl esters (δ 3.80) and acetyl groups (δ 2.17, 2.06). The polysaccharide had a 6.0 × 10^4^ g/mol molar mass and a 26.2 mol% degree of esterification, with triads of esterified GalA (EEE, 43%) dominating over partially esterified regions. FTIR confirmed ester carbonyl (1740 cm^−1^) and carboxylate (1653 cm^−1^) bands, whereas ^13^C NMR confirmed anomeric carbons of GalA (δ 101.1), Araf (δ 108.5), and Rha (δ 17.5), all pointing to a structurally diverse polysaccharide adapted to tropical biotic interactions [146].

Mangaba (*Hancornia speciosa*), which is found in Brazilian tropical savannas, grows in well-drained soil under seasonal drought conditions [147]. Soluble (SDF) and insoluble (IDF) dietary fiber fractions of the fruit were isolated enzymatically, and Ara-rich polysaccharides were isolated. SDF was composed of 46.3% Ara, 24.2% Gal, and traces of Xyl, Man, Glc, and Rha, along with 5% uronic acids. IDF, after alkaline solubilization (IDF-KS), was composed of 75% Ara, 6.3% Gal, and 14.5% uronic acids, predominantly GlcA. Structural analyses revealed (1→5)-linked α-L-Ara*f* units in both fractions, a characteristic of arabinans found in RG-I from pectic networks. IDF-KS also showed (1→4)-linked β-D-Gal*p* residues, a feature of type I arabinogalactans. HPSEC showed SDF to be a heterodisperse mixture containing three discrete molar mass populations, whereas IDF-KS contained a single broad peak. NMR corroborated the presence of esterified lignin signals (δ 53.4/4.34) in IDF-KS and non-carbohydrate constituents (latex-derived polyisoprenes) in SDF. The arabinan-rich structure, including RG-I domains and uronic acid composition, reflects responses to water retention and resilience to mechanical stress in arid-adapted fruits [148].

The convergences and divergences between the fruits’ bioactive polysaccharides and their glycans in specific biomes are found below, in Table 10.

## 4. Global Diversity of Polysaccharide-Rich Fruits: A Cross-Biome Synthesis

**Figure 3:**
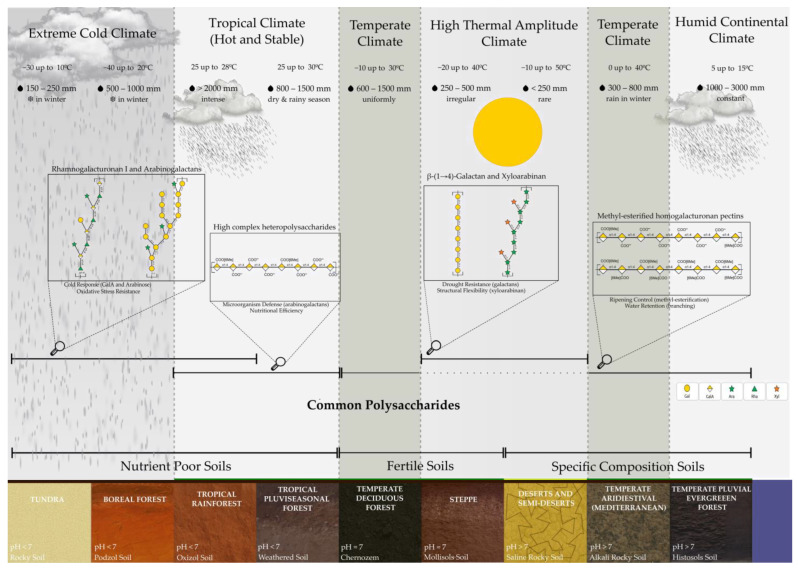
Correlation between abiotic components (pluviosity, climate, soil) and the main groups of glycans present in edible fruits. Drawings generated by the Inkscape v. 1.4 software.

Fruit polysaccharide chemical composition evolves from a long and involved past, based on abiotic and biotic factors, to confer resistance to unfavorable elements that act upon their reproduction strategy. The monomer content levels and chemistry of polysaccharides reveal special adaptations within an environment and indicate ecological hardiness under extremely diverse conditions (Figure 3) [149].

Soil is a fundamental element that affects the strategies of the mentioned species, which also determine the characteristics of their respective fruits [150]. The nine biomes of Tundra, Boreal Forests, and Tropical Forests are characterized by having slightly acidic soils with a pH below 6.5, a condition linked to high acidic polysaccharides, including pectins with a high GalA content, which are responsible for low-temperature stress tolerance [47].

Conversely, alkaline soils prevalent in desert/arid and Mediterranean biota with pH levels above 7.5 support vegetation that contains high de-esterified polysaccharide levels and/or significant galactan levels. This is prompted by the natural necessity to guard against water stress by forming protective layers to negate evapotranspiration [151].

In deciduous and steppe biomes, fertile soils that promote deep roots and neutral pH, combined with nutrient concentrations from organic matter richness, including inherent calcium, magnesium, phosphorus, and potassium ions, favor the evolution of fruits with controlled ripening. This is due to methylated pectins and a very controlled calcium uptake process [152].

In tropical forests, where nutrient-poor, acidic soils (pH < 5.0) and high surface organic matter deposition coexist with exposed and large-diameter roots, arabinogalactans are present in significant quantities to form symbiotic associations with mycorrhizae, thereby creating a high availability of nutrients for the effective growth of reproductive organs [153]. In environments with a high density of alkaline earth minerals, such as deciduous forests, some Mediterranean ecotones, and steppes, the fruit’s molecular composition is highly conditioned. This characteristic not only reinforces protection mechanisms against pathogens but also, in conjunction with a progressive ripening process, enables better adaptation of species to summer aridity [154].

Molecular mechanisms associated with the high calcium availability in the soils of these biomes involve the formation of organic acid salts that contribute to pectin rigidity, and the regulation of pectin methylesterase enzymes to manage fruit firmness [155]. Extreme salinity, especially of desert soils due to high evaporation levels, presents a host of difficulties that are key to identifying indigenous species adaptations [156]. Date palms, for instance, exhibit increased production of proline and glycine betaine in their proteins, shielding membranes against osmotic stress in water-scarce, salt-laden soils. *Opuntia ficus-indica* galactans enables intracellular saline regulation by allowing water retention within the mesocarp [157].

In rainforests and biomes with podzolic or frozen soils, high levels of ferric, ferrous, and aluminum ions lead to severe selective pressure on angiosperms, triggering adaptations that enable tolerance to metal toxicity [158]. Fruits like cranberries have high levels of GalA in their pectins, which facilitates the creation of metal complexes with aluminum ions and mitigates toxicity. The high occurrence of RGs also has a central role in managing metal toxicity through sequestering aluminum ions into vacuolar compartments [65,78].

The climatic and soil conditions have a direct influence on the polysaccharide composition of native fruits, including the content of polysaccharides, their main constituent monomers, solubility, and related functions. High GalA and Ara content polysaccharides are predominant in the Tundra and Boreal Forest biomes, where they play direct roles in cold tolerance (as antifreeze substances) and in water retention in the freezing soils [159]. High levels of α-(1→4) and β-(1→4) glycosidic bond frequencies, typical for polysaccharides like amino-cellulose, are hallmarks of structural adjustments to make fruits resilient in the inclement climates of these biomes. This holds within it free monosaccharides, which are vital for rapid germination activities and the characteristic sweetness required for reproductive dispersal via avifauna [160].

Both high concentrations of GalA and Ara in the fruits of Deciduous and Temperate Rainforest biomes are also related to higher fruit durability. However, highly methylated pectins in the fruits of Deciduous Forests (strawberries and rose hips, for example) and compact, soluble polysaccharides in the fruits of Rainforests (persimmons and fig fruits, for example) provide such fruits with better regulation of the ripening process and quicker nutrient assimilation, respectively [81,82,87,88,94,95,97]. Although α-(1→4) and β-(1→4) bonds are still prevalent, uncommon glycosidic bonds in fruits, such as cherries (α-(1→5)) and blackberries and fig fruits (β-(1→3,6)), can be associated with greater plasticity in humid climates or flexibility throughout the seasons.

Despite the general differences, the Steppe, Mediterranean, and Desert biomes share a common correlation between high de-esterified pectin and arabinan, xyloarabinans, and arabinogalactans, which contribute to drought resistance, UV protection, and mucilage structuring, thereby facilitating precise evapotranspiration regulation. Steppe and Desert biomes also have a high Rha and Gal composition, which strengthens the plant parenchymal tissue and regulates the structural matrices of fruits. This indicates a high energy investment by these fruits in retaining water, giving them succulence, despite the water- and nutrient-deficient soil [161,162].

Seasonal and Humid Tropical Forests are characterized by GalA- and Ara-prevailing polysaccharides with rich galactan portions in pectic frameworks. Fruits from these biomes show advanced pectin networks with dynamic ripening processes. As in papaya fruit, a fast ripening is associated with a massive enzymatic action in HG and RG-I degradation, while mango fruits employ RG-I and arabinogalactans as storage polysaccharides [129,130,131].

In the cold biomes of the Tundra and Boreal Forests, the significant occurrence of GalA, often conjugated to phenolics, illustrates a role associated with highly efficient freeze-stress protection. This is facilitated by the water content in fruits and the suppression of oxidative activity by phenolic moieties, which neutralize free radicals caused by extreme cold. Consistent with this, the polysaccharide characteristics of berries in these biomes prioritize rapid germination in warmer seasons and structural integrity, regardless of freezing conditions [163].

In humid tropical forests, esterified pectins play a crucial role in anti-pathogenic activity by forming physical barriers with strong effectiveness against a broad spectrum of microorganisms. The extensive branching of arabinogalactan polysaccharides also defends fruits by suppressing biotic stress. Polysaccharides and derivatives of monosaccharides also serve as key factors in adaptability, primarily through seed dispersal by frugivores [164].

Across biomes, environmental pressures such as temperature extremes, water limitation, and nutrient availability shape how fruit polysaccharides are assembled. In tundra and boreal forests, where low temperatures and frozen soils prevail, cell walls tend to form compact, gel-like matrices that are especially rich in uronic acids and homogalacturonan regions.

These structures, characterized by relatively high levels of galacturonic acid and arabinose, moderate methylation (DM around 50–75%), and few side branches, help prevent cellular damage during freezing by stabilizing water and ion distribution within the wall. The main result is a denser, less permeable matrix that stabilizes integrity during prolonged cold periods.

On the other hand, fruits from arid and semi-arid biomes (Mediterranean, steppe, deserts) exhibit polysaccharide diversification, characterized by higher branching, acetylation, and arabinogalactan side chains, which confer hydrophilicity and elasticity under water-deficit conditions. The predominance of xyloarabinan, galactan, and arabinogalactan motifs increases water retention. High acetylation of xylans and low degrees of methyl esterification facilitate osmotic buffering and enzymatic resistance, sustaining hydration cycles during drought.

In tropical and pluviseasonal biomes, constant warmth and high rainfall promote highly substituted, flexible pectic networks that combine HG and RG-I/RG-II domains with arabinan and galactan side chains. These structures support cell wall porosity, softening, and dynamic remodeling during ripening—traits favored in continuously growing species. Together, polysaccharide architectures shift along environmental gradients: from linear, cryo-stable HG networks in freezing biomes to branched, hydrophilic RG-enriched matrices in drought-prone environments, and to highly esterified, dynamic pectins in humid tropics. Such structural motifs reflect adaptive strategies optimizing water retention, thermal buffering, and mechanical flexibility under biome-specific stresses.

Despite convergent features, fruits within each biome can exhibit alternative adaptations associated with distinctive developmental characteristics. Therefore, this refers to the profound impact of the intricate polysaccharide networks in native edible fruits, as well as the environmental pressures and specificities of each biome. Thus, polysaccharides are not only essential molecules for the full development of the mentioned species but also accurate chemical fingerprints of these ecological interactions.

## 5. Conclusions and Future Perspectives

Polysaccharides are biologically advanced molecules with heterogeneous structures and play a crucial role in the cellular and molecular processes of fruits. They play a role in driving development and also function in evolutionary interactions that lead to seed dispersal through the multiplication of species. Their bioactivity extends beyond the well-explored areas of cell wall structure and metabolism in endemic and native fruits, especially when it directly correlates with soil type, climate, and neighboring biotic and abiotic features.

Specifically in edible fruits, the structural and monosaccharide profiles of bioactive polysaccharides consistently reflect the dominant abiotic pressures of the biome of origin. Measurements of galacturonic acid composition, hexose/pentose ratio, degree of esterification, molecular mass, and degree of branching, among others, provide methods for characterizing these important chemical signatures, which converge with abiotic factors such as average and seasonal temperatures, water regimes, and soil characteristics. Therefore, they are not purely descriptive characteristics. They imply functional and structural properties that reflect the molecular and metabolic functions of the fruit, its water activity and consequent water retention, freezing resistance, and the solubility and temporal availability of metabolites and substrates that make the fruit’s phenotype suitable for its biological functions and that are used in human food cultures of different ethnicities and locations.

In the present review, convergent patterns were identified across biomes, supporting functional hypotheses of both convergence and divergence. In cold environments, pectins appear with a higher relative content of galacturonic acid and chains rich in arabinans, characteristics that favor gelation at low temperatures and stabilization of the cell wall in freezing situations; in arid biomes, there is a prevalence of xyloarabinans and galactans with greater hydrophilic characteristics, suggesting a role in water retention and maintenance of tissue plasticity under conditions of water stress and water scarcity. In environments with high water variability, greater branching, the presence of acetyl groups, and a lower degree of esterification were observed, changes that, combined, increase viscosity and slow water loss.

When extrapolated, such characteristics can provide a valuable molecular landscape that, consequently, can be used both to achieve a deeper understanding of how the physicochemical factors of the environment directly influence the different species of edible fruits existing on the planet—with smaller, non-climacteric fruits appearing in colder regions, while larger, fleshier fruits are typical of tropical climates and exhibit climacteric behavior, despite nuances in this issue, which are precisely related to variations in monosaccharides fractions extensively discussed here—and for quantitative use in fruit breeding methods, thereby providing glycosidic characteristics that enhance resistance to cold, heat, water stress, soil acidity or alkalinity, among others.

Furthermore, it is vital to highlight the methodologies for extracting and quantifying bioactive polysaccharides used in each important article discussed here. Differences in extraction protocols and analytical techniques, such as comparing articles that used acid hydrolysis followed by chromatography with those that used Nuclear Magnetic Resonance, are significant factors that limit the narrative research presented here. These differences provide a basis for new analyses based on this article, addressing not only biomes but also precise locations, fruit stages, homogeneous extraction methods, and analytical parameters for both species and soil. It is also important to emphasize the integration of omics fields, especially glycomics and transcriptomics, as well as phenotypic assays under stress conditions, into the bioactive polysaccharide study area, thereby increasing the homogeneity of results and expanding the associated molecular network.

Therefore, in the context of edible fruits, polysaccharides are not restricted to traditional structural roles but also serve as multifunctional biomolecules whose ecological significance extends to ecosystem-level processes. This perspective underscores the importance of cross-disciplinary approaches that bridge molecular mechanisms with macroecological dynamics, allowing a more integrated understanding of polysaccharides and their roles in plant adaptation and food systems.

## Figures and Tables

**Table 1:** Summary of the Tundra Biome.

Fruits	General Functional Implications
*Vaccinium uliginosum* (Arctic blueberry)	Cryoprotective polysaccharides aiding water stabilization and freeze tolerance.
*Vaccinium vitis-idaea* (Lingonberry)	Polyphenol–polysaccharide conjugates providing antioxidant defense and cold adaptation.
*Vaccinium oxycoccos* (Cranberry)	RG-I–rich pectins improving gelation and resilience to freezing.
*Rubus chamaemorus* (Cloudberry)	Free monosaccharides supporting osmotic regulation under low temperature.
*Arctostaphylos uva-ursi* (Bearberry)	Phenolic-linked polymers contributing to stress resistance (leaves documented).

**Table 2:** Summary of the Boreal Forest Biome.

Fruits	General Functional Implications
*Vaccinium myrtillus* (Bilberry)	Acetylated xylans and methylated pectins increasing rigidity and cold endurance.
*Sorbus aucuparia* (Rowanberry)	Arabinan–polyphenol interactions providing antioxidant buffering.
*Rubus chingii* (Asian raspberry)	Branched RG-I polysaccharides conferring flexibility and cold tolerance.
*Vaccinium macrocarpon* (Cranberry)	HG and RG-I pectins balancing mechanical strength and water retention.

**Table 3:** Summary of the Temperate Deciduous Forests.

Fruits	General Functional Implications
*Fragaria ananassa* (Strawberry)	Highly methylated HG pectin maintaining firmness during ripening.
*Prunus avium* (Sweet cherry)	Acidic heteropolysaccharides modulating texture and antioxidant potential.
*Rubus idaeus* (Raspberry)	Glucan- and arabinogalactan-rich polymers enhancing stress response.
*Morus nigra* (Mulberry)	Uronic acid–rich polysaccharides with hypoglycemic and antioxidant activity.
*Rosa canina* (Rose hip)	HG–RG-I hybrid pectin with antioxidant and immunomodulatory effects.

**Table 4:** Summary of the Temperate Pluvial Evergreen Forest Biome.

Fruits	General Functional Implications
*Persea americana* (Avocado)	Mannoheptulose and perseitol aiding osmoregulation and postharvest metabolism.
*Citrus sinensis* (Orange)	HG–RG-I pectin matrix enhancing hydration and tissue firmness.
*Diospyros kaki* (Persimmon)	Polysaccharide heterogeneity supporting water balance and nutraceutical potential.
*Ficus carica* (Fig fruit)	Branched heteropolysaccharides contributing to hydration and soft texture.

**Table 5:** Summary of the Aridiestival Evergreen Forests Biome.

Fruits	General Functional Implications
*Olea europaea* (Olive)	De-esterified pectins and xyloglucans maintaining firmness and drought tolerance.
*Prunus amygdalus* (Almond)	Ara- and Gal-rich pectins reinforcing structure under arid stress.
*Punica granatum* (Pomegranate)	Highly esterified HG pectins providing antioxidant protection and stability.
*Arbutus unedo*	Lignified xylan–cellulose networks ensuring drought resistance and firmness.

**Table 6:** Summary of the Steppe Biome.

Fruits	General Functional Implications
*Hippophae rhamnoides* (Sea buckthorn)	HG pectins and arabinogalactans enhancing heat and drought tolerance.
*Berberis vulgaris* (Barberry)	Pectin heteropolymers supporting antioxidant and mechanical stability.
*Lycium barbarum* (Goji)	RG-I and HG fractions mediating osmotic protection and immunomodulation.
*Prunus armeniaca* (Apricot)	RG-I–rich pectins improving solubility and stabilizing bioactivity.
*Nitraria retusa*	Branched arabinogalactans aiding salt tolerance and antioxidant defense.

**Table 7:** Summary of the Deserts and Semi-Deserts Biome.

Fruits	General Functional Implications
*Opuntia ficus-indica* (Prickly pear)	Mucilaginous arabinogalactans enabling water retention and desiccation resistance.
*Ziziphus jujuba* (Jujube)	RG-I pectins and arabinans contributing to drought adaptation and antioxidant activity.
*Citrullus lanatus* (Watermelon)	Branched RG-I and arabinogalactan networks ensuring hydration control.
*Phoenix dactylifera* (Date palm)	Hemicellulosic xylans and galactomannans strengthening seed structure and water balance.

**Table 8:** Summary of the Tropical Pluviseasonal Forests Biome.

Fruits	General Functional Implications
*Carica papaya* (Papaya)	Dynamic HG–RG-I remodeling regulating softening and enzymatic ripening.
*Mangifera indica* (Mango)	Arabinogalactan–RG complex ensuring cell wall flexibility and antioxidant defense.
*Psidium guajava* (Guava)	Ara- and Gal-rich heteropolysaccharides improving stress tolerance and digestibility.
*Anacardium occidentale* (Cashew)	Branched β-D-galactans conferring hydration stability and antimicrobial barrier.
*Hylocereus undatus* (Dragon fruit)	Acidic heteropolysaccharides maintaining cell wall integrity in dry habitats.

**Table 9:** Summary of the Tropical Rainforests Biome.

Fruits	General Functional Implications
*Euterpe oleracea* (Açaí)	Arabinogalactans and pectins providing elasticity and antioxidant protection.
*Ananas comosus* (Pineapple)	Man-rich heteropolysaccharides aiding tissue flexibility and enzymatic defense.
*Theobroma grandiflorum* (Cupuaçu)	HG–RG-I pectins supporting water regulation and pathogen resistance.
*Passiflora edulis* (Passion fruit)	Low-esterified pectins modulating viscosity and antimicrobial defense.
*Hancornia speciosa* (Mangaba)	Pectic polysaccharides with arabinogalactan branches contributing to high water retention, antioxidant activity, and cell wall elasticity under humid conditions.

**Table 10:** Composition of glycans in the native edible fruits found in each biome.

Biomes	Fruits	Identified Polysaccharides	Structure	Ref.
Tundra	Arctic Blueberry (*Vaccinium* *uliginosum*)	VUP-1 (heteropolysaccharide)	Ara, Man, GalA, Glc and Gal	[62]
	Lingonberry (*Vaccinium vitis-idaea*)	Acidic polymers and neutral arabinogalactans esterified with hydroxycinnamates	Glc, Ara, Gal, GalA, GlaA, alongside trace amounts of Rha, Fuc, Xyl, Rib	[63,64]
	Cranberry (*Vaccinium oxycoccos*)	RG-I pectic polysaccharides	GalA, homogeneous HG domains, branched RG-I with arabinogalactan side chains	[65,66]
	Cloudberry (*Rubus chamaemorus*)	No structural polysaccharides have been characterized to date	Carbohydrate fraction: Gly, Fru, Xyl, Gal and Ara	[67,68]
	Bearberry (*Arctostaphylos* *uva-ursi*)	No structural polysaccharides have been characterized to date	-	[69]
Boreal Forest	Bilberry (*Vaccinium myrtillus*)	Glc-rich HCs, cellulose and low pectin constitution	HG with methyl esterification; RG-I with arabinan side chains	[73,74]
	Rowanberry (*Sorbus aucuparia*)	Water-soluble pectin	High GalA; HG backbone + RG-I domains; Ara*f* and Gal*p* side chains	[73,74]
	Raspberry (*Rubus chingii*)	Acidic heteropolysaccharide (pRCP)	Backbone of →3,6)-β-D- Gal*p* + →5)-α-L-Ara*f*; Ara (39.76%) and Gal (39.43%)	[75,76]
	Cranberry (*Vaccinium macrocarpon*)	Stratified pectic polysaccharides	Methyl-esterified HG (75%) + arabinan/galactan side chains; RG-I with arabinogalactan substitutions (Ara + Gal/Rha = 11.5:1)	[77,78]
Temperate Deciduous Forests	Strawberries (*Fragaria ananassa*)	Pectins (GalA-rich) and HCs	High methylation (60%); HG-dominated regions with arabinogalactan side chains	[80,81,82]
	Sweet cherries (*Prunus avium*)	Ara/Gal-rich heteropolysaccharides	Ara, Gal, Glc and uronic acids	[83,84]
	Raspberries (*Rubus idaeus*)	Ara-rich polysaccharides	(1→4)-α-glucans; enzyme-resistant RG-I fragments	[84]
	American ginseng berries (*Panax quinquefolius)*	Gal-rich heteropolysaccharides	Low uronic acids; protein-polysaccharide interactions	[84]
	Mulberry (*Morus nigra*)	Ultrasound-assisted extracted polysaccharides	Glc, Ara, Gal and uronic acid	[85,86]
	Rose hip (*Rosa canina*)	GalA-rich pectin	HG backbone with methylesterification and acetylation, with RG-I segments; unique oligomers, including unsaturated pentamers with dual methyl and acetyl substitutions	[87,88]
Temperate Pluvial Evergreen Forest	Avocado (*Persea americana*)	Pectin	Cellulose/hemicellulose, pectin; C7 sugars (mannoheptulose, perseitol)	[90,91]
	Orange (*Citrus sinensis*)	Methyl-esterified HG + RG-I	Albedo pectin (83–85% GalA); branched RG-I with Ara/Gal side chains	[92,93]
	Persimmon (*Diospyros kaki*)	Water-soluble polysaccharides	Ara-rich side chains, Gal, Glc and GalA	[94,95]
	Fig fruit (*Ficus carica*)	Heteropolysaccharides	Ara, Gal, Gly; Branched α-1,4/β-1,3,6 linkages; HG (pectin) + xyloglucans (hemicellulose)	[96,97]
Temperate Aridiestival Evergreen Forests	Olive (*Olea europaea*)	HG + RG-I pectins	Reduced methyl esterification/acetylation during ripening; increased RG-I branching	[99,100]
	Almond (*Prunus amygdalus*)	Pectic polysaccharides	Peel: HG and RG-I domains; HCl-soluble pectin; GalA, Ara, Gal, Man, acid uronic. Seed: Ara-rich, xyloglucans and acidic xylans embedded in cellulose-hemicellulose matrix	[101,102,103]
	Pomegranate (*Punica granatum*)	Pectic polysaccharides	HG pectins: 46–68% GalA; high methylation + acylation; branched Ara (1→5)-α-L-Ara*f*	[104,105,106]
	*Arbutus unedo*	Cellulose + HCs (xylans/xyloglucans)	Glc, Xyl and Ara; Lignified matrix with scleroids; reduced HG esterification during ripening	[107,108]
Steppe	Sea Buckthorn (*Hippophae Rhamnoides*)	Hot water, ultrasonic-, microwave- and ethanol-assisted extracted polysaccharides	GalA, Glc, Gal, Ara, Rha, Xyl and Man; β-(1→4)-galactan backbone and α-(1→5)-Ara*f* with Xyl substitutions	[111]
	Barberry (*Berberis vulgaris*)	Pectic polysaccharides	High GalA, Ara/Gal-dominated side chains; branched RG-I + HG domains	[110,111]
	Goji (*Lycium barbarum*)	Pectic polysaccharides	Alkali-extracted RG-I (Ara + Gal/Rha = 7.77); acid-extracted HG with low branching	[112,113]
	Apricots (*Prunus armeniaca*)	Pectic polysaccharides	RG-I: Ara, Gal; Arabinogalactan-protein conjugates; high Man in hemicellulose	[114]
	*Nitraria retusa*	Heteropolysaccharides	Ara, Gal, Glc; α-(1→6)-galactan + branched arabinan; antioxidant activity linked to uronic acids	[115,116]
Deserts and Semi-Deserts	Prickly pear (*Opuntia ficus-indica*)	Heteropolysaccharides	Linear β-(1→4)-galactan backbone; complex α-arabinan with Xyl substitutions. (α-(1→5)-arabinan with 2,3,5-linked branches.)	[118,119]
	Jujube (*Ziziphus jujuba*)	RG-I (arabinogalactan) + arabinan	α-(1→4)-GalA backbone + β-(1→4)-galactan/α-(1→5)-arabinan side chains.	[120,121]
	Watermelon (*Citrullus lanatus*)	RG-I pectin (arabinogalactan)	β-(1→6)-galactan + α-L-Ara*f* side chains; compact branched conformation	[122,123,124]
	Date palm (*Phoenix dactylifera*)	Heteropolysaccharides	Mesocarp: (1→3)-β-D-Glc*p* backbone with (1→6)-linked branches; Seed xylan: β-(1→4)-Xyl*p* with Ara/Gal substitutions	[125,126]
Tropical Pluviseasonal Forests	Papaya (*Carica papaya*)	Pectic polysaccharides	HG pectins: High GalA (69–74%), reduced RG-I during ripening; β-glucans released from cellulose during ripening	[128,129]
	Mango (*Mangifera indica*)	Heteropolysaccharides	Arabinogalactan, RG; 1→4-β-galactan backbone + 1→5-α-Ara*f*	[130,131]
	Guava (*Psidium guajava*)	Heteropolysaccharides	Ara-rich, Glc; (1→5)-α-L-Ara backbone; branched (1→3,6)-β-D-Gal	[132,133]
	Cashew (*Anacardium occidentale*)	Branched heteropolysaccharides	β-(1→3,6)-Gal*p*; side chains with GlcA, Ara, Rha	[134]
	Dragon fruit (*Hylocereus undatus*)	Heteropolysaccharides	GlcA, Gal, Rha; backbone: →4-β-GlcA + →6-β-Gal; side chains: α-L-Ara*f*-(1→5)-α-L-Ara*f*	[135,136]
Tropical Rainforests	Açaí (*Euterpe oleracea*)	Arabinogalactan + HG pectin	β-(1,3)-galactan backbone + Ara/Gal side chains; partial esterification	[138,139,140]
	Pineapple (*Ananas comosus*)	Man-rich heteropolysaccharides	Ara, Xyl, Man, Glc, Gal, Rha, GalA; →4)-α-D-Man*p* backbone; Ara/Gal side chains + methyl-esterified GalA	[141,142]
	Cupuassu (*Theobroma grandiflorum*)	Pectic polysaccharides	High methyl esterification, low acetylation; RG-I backbone contained →4)-α-D- Galactopyranosyl Acid (GalA*p*)-(1→ and →2,4)-α-L-Rha*p*-(1→ linkages, substituted at O-4 of Rha with side chains rich in Gal and Ara	[143,144]
	Passion fruit (*Passiflora edulis*)	Pectic polysaccharides	GalA, Ara, Rha; (1→4)-GalA backbone; low esterification + acetyl groups.	[145,146]
	Mangaba (*Hancornia speciosa*)	Heteropolysaccharides	Arabinan (RG-I-associated); (1→5)-α-L-Ara*f* units; (1→4)-β-D-Gal*p* residues.	[147,148]

## Data Availability

No new data were created or analyzed in this study. Data sharing is not applicable to this article.

## Abbreviations

The following abbreviations are used in this manuscript:

AFM	Atomic Force Microscopy
AISs	Alcohol-Insoluble Solids
Ara	Arabinose
Araf	Arabinose-furanose
CAM	Crassulacean Acid Metabolism
CDTA	Chelating Agent
DA	Degree of Acetylation
DEAE cellulose	Diethylaminoethyl cellulose
DM	Degree of Methylation
EDTA	Ethylenediaminetetraacetic Acid
Fru	Fructose
FT-IR	Fourier Transform Infrared Spectroscopy
Fuc	Fucose
Gal	Galactose
GalA	Galacturonic acid
GalA*p*	Galactopyranosyl Acid
Gal*p*	Galactopyranose
Gal*p*A	Galactopyranose Acid
GC/MS	Gas Chromatography–Mass Spectrometry
Glc	Glucose
GlcA	Glucuronic acid
Glc*p*	Glucose-pyranose
GlcpA	Glucuronic-pyranose Acid
HC	Hemicellulose
HCl	Hydrochloric Acid
HG	Homogalacturonan
HPAEC-PAD	High-Performance Anion-Exchange Chromatography with Pulsed Amperometric Detection
IDF	Insoluble Dietary Fiber
KOH	Potassium Hydroxide
Man	Mannose
Man*p*	Mannose-pyranose
Me	Methyl
MW	Molecular Weight
Na_2_CO_3_	Sodium Carbonate
NaOAc	Sodium Acetate
NMR	Nuclear Magnetic Resonance
NOE	Nuclear Overhauser Effect
ORAC	Oxygen Radical Absorbance Capacity
PAPs	Pineapple Polysaccharides
PeSs	Pectin Substances
PS-I	Heteropolysaccharide-I of *P. guajava*
RG	Rhamnogalacturonan
Rha	Rhamnose
Rha*p*	Rhamnose-pyranose
Rib	Ribose
SEC-MALLS	Size Exclusion Chromatography coupled with Multi-Angle Light Scattering
USA	United States of America
VUP-1	*Vaccinium uliginosum* polysaccharide
WSPs	Water-Soluble Polysaccharides
Xyl	Xylose
Xyl*p*	Xylose-pyranose
